# Digital Light 3D Printing of PEDOT-Based Photopolymerizable Inks for Biosensing

**DOI:** 10.1021/acsapm.2c01170

**Published:** 2022-08-10

**Authors:** Naroa Lopez-Larrea, Miryam Criado-Gonzalez, Antonio Dominguez-Alfaro, Nuria Alegret, Isabel del Agua, Bastien Marchiori, David Mecerreyes

**Affiliations:** † POLYMAT, University of the Basque Country UPV/EHU, Paseo Manuel de Lardizabal 3, 20018 San Sebastián, Spain; ‡ Carbon Bionanotechnology Group, Center for Cooperative Research in Biomaterials (CIC biomaGUNE), Basque Research and Technology Alliance (BRTA), 20014 San Sebastian, Spain; § IIS Biodonostia, Neurosciences Area, Group of Neuromuscular Diseases, Paseo Dr. Begiristain s/n, 20014 San Sebastian, Spain; ∥ Panaxium SAS, Aix-en-Provence 13100, France; ⊥ Ikerbasque, Basque Foundation for Science, 48013 Bilbao, Spain

**Keywords:** conducting polymers, photopolymerizable inks, hydrogels, digital light printing, biosensing

## Abstract

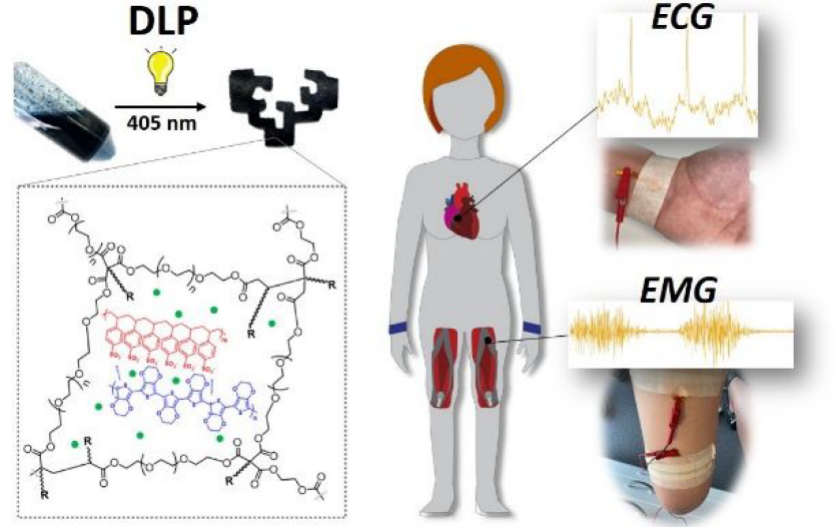

3D conductive materials such as polymers and hydrogels that interface between biology and electronics are actively being researched for the fabrication of bioelectronic devices. In this work, short-time (5 s) photopolymerizable conductive inks based on poly(3,4-ethylenedioxythiophene) (PEDOT):polystyrene sulfonate (PSS) dispersed in an aqueous matrix formed by a vinyl resin, poly(ethylene glycol) diacrylate (PEGDA) with different molecular weights (*M*_n_ = 250, 575, and 700 Da), ethylene glycol (EG), and a photoinitiator have been optimized. These inks can be processed by Digital Light 3D Printing (DLP) leading to flexible and shape-defined conductive hydrogels and dry conductive PEDOTs, whose printability resolution increases with PEGDA molecular weight. Besides, the printed conductive PEDOT-based hydrogels are able to swell in water, exhibiting soft mechanical properties (Young’s modulus of ∼3 MPa) similar to those of skin tissues and good conductivity values (10^–2^ S cm^–1^) for biosensing. Finally, the printed conductive hydrogels were tested as bioelectrodes for human electrocardiography (ECG) and electromyography (EMG) recordings, showing a long-term activity, up to 2 weeks, and enhanced detection signals compared to commercial Ag/AgCl medical electrodes for health monitoring.

## 1 Introduction

Wearable electronic devices attract great attention in several fields covering a wide range of applications, including sensors, soft robotics, touch panels, healthcare monitoring, and artificial electronic skin.^1−6^ In particular, the development of cutaneous electronic devices able to measure human biopotentials represents a noninvasive powerful tool for the assessment of physiological and pathological signals allowing detection and prevention of diseases at early stages.^7−10^ In this regard, the discovery of materials with robustness, flexibility, and electrical stimulus sensitivity that create an accurate interface between biology and electronics is actively being researched.^11−13^

Conducting polymers (CPs), i.e., poly(3,4-ethylenedioxythiophene) (PEDOT), polypyrrole (PPy), and polyaniline (PANi), with intrinsic electronic properties and soft nature, compared with traditional electronic inorganic fillers, have been investigated for such bioelectronic applications.^14,15^ Among them, PEDOT is the most widely used CP due to its high electrical conductivity, thermal stability, and biocompatibility.^16^ However, its infusibility and water insolubility limit its processability to traditional two-dimensional (2D) thin films by solvent-casting or spin-coating methods. Thus, PEDOT has usually been used as a filler blended with other polymers for the fabrication of 2D PEDOT-based devices with electronic properties. In some cases, this immiscible filler tends to aggregate, resulting in the conductive paths’ percolation. This impedes the adaptive movement of the conductive network during stretching, affecting the electronic properties.^17^ Moreover, 2D PEDOT-based devices are usually physically and mechanically different from biological tissues that contain large amounts of water and have soft mechanical properties, with Young’s moduli in the range of 1 kPa–1 MPa^18^

For such purpose, new PEDOT-based polymer formulations that enhance PEDOT processability by 3D printing techniques have been investigated.^19,20^ Very recently, we have shown that PEDOT can be copolymerized with different biopolyesters, i.e., poly(ε-caprolactone) (PCL) and poly(lactic acid) (PLA), leading to graft copolymers, PEDOT-*g*-PCL and PEDOT-*g*-PLA, respectively, with excellent shear-thinning behavior to be processed by direct ink writing (DIW) in the melting state.^21^ These 3D scaffolds showed biocompatibility in the presence of myoblasts and cardiomyocytes, as well as electroactive properties for tissue engineering applications.^21,22^ Apart from that, the development of printable conductive hydrogels with enhanced flexibility and adhesion properties would be beneficial.^23,24^ That is the case of PEDOT:polystyrene sulfonate (PSS) dispersions mixed with organic solvents, poly(vinyl alcohol) (PVA), or natural polymers forming shear-thinning gels (10^2^–10^3^ Pa s) able to be printed by DIW in a shape-defined three-dimensional (3D) structure to be employed as soft sensors or cell-laden scaffolds.^25−27^ Besides, PEDOT:PSS dispersions have also been inkjet-printed for the fabrication of organic electrochemical transistors (OECTs) and conformable tattoo electrodes for electrophysiology.^28−30^ Likewise, inkjet-printing was employed to print photocurable formulations of PEDOT:PSS, 2-cholinium lactate methacrylate, ethylene glycol dimethacrylate as a cross-linker, and a photoinitiator, which required a postprinting photopolymerization step to obtain the final patterns for electrocardiography (ECG), making this process time-consuming.^10^ This drawback can be solved by using light-based printing technologies such as stereolithography (SLA) and digital light printing (DLP), which present high-resolution printing, ∼100 μm, as compared with DIW and inkjet-printing.^31,32^ This specific feature becomes highly attractive for the fabrication of medical devices, which need a high shape fidelity.^33,34^ Furthermore, as it is based on the photopolymerization of a monomer or a mixture of monomers, the design of an ink formulation able to photocure in short periods during the layer-by-layer printing of the piece plays a key role.^35^ As an emerging technique in the field of conducting polymers, few works employing light-based printing are reported in the literature. In this way, SLA was used by Zhang and co-workers to photoprint PEDOT-based gelatin methacryloyl (GelMA) hydrogels that were used for neuronal differentiation.^36^ PEDOT-based photopolymerizable inks have also been developed by mixing PEDOT:PSS with poly(ethylene glycol) diacrylate (PEGDA) and a photoinitiator to be processed by SLA. The dispersion of PEDOT in the photocurable PEGDA matrix was favored by acid treatment in concentrated sulfuric acid, and the 3D printed materials were used as organic electrochemical transistors (OECTs).^37−39^ However, as SLA is limited to a single spot photopolymerization while printing, DLP that prints a continuous plane allows accelerating the printing process while retaining a high-quality resolution. Very recently, Shao and co-workers employed a different methodology to favor the PEDOT dispersion in the PEGDA matrix. In that case, the commercial PEDOT:PSS aqueous solution was freeze-dried and dispersed in a water/ethanol mixture. Moreover, the incorporation of silica nanoparticles into the inks allowed researchers to obtain shape-defined electrodes by DLP to be used in lithium-ion batteries (LIBs).^40^ Thus, the discovery of photocurable and electroactive polymer inks that can be processed by digital light 3D printing to fabricate flexible and wearable electronic devices with tailor-made shapes remains a challenge.

**Scheme 1:**
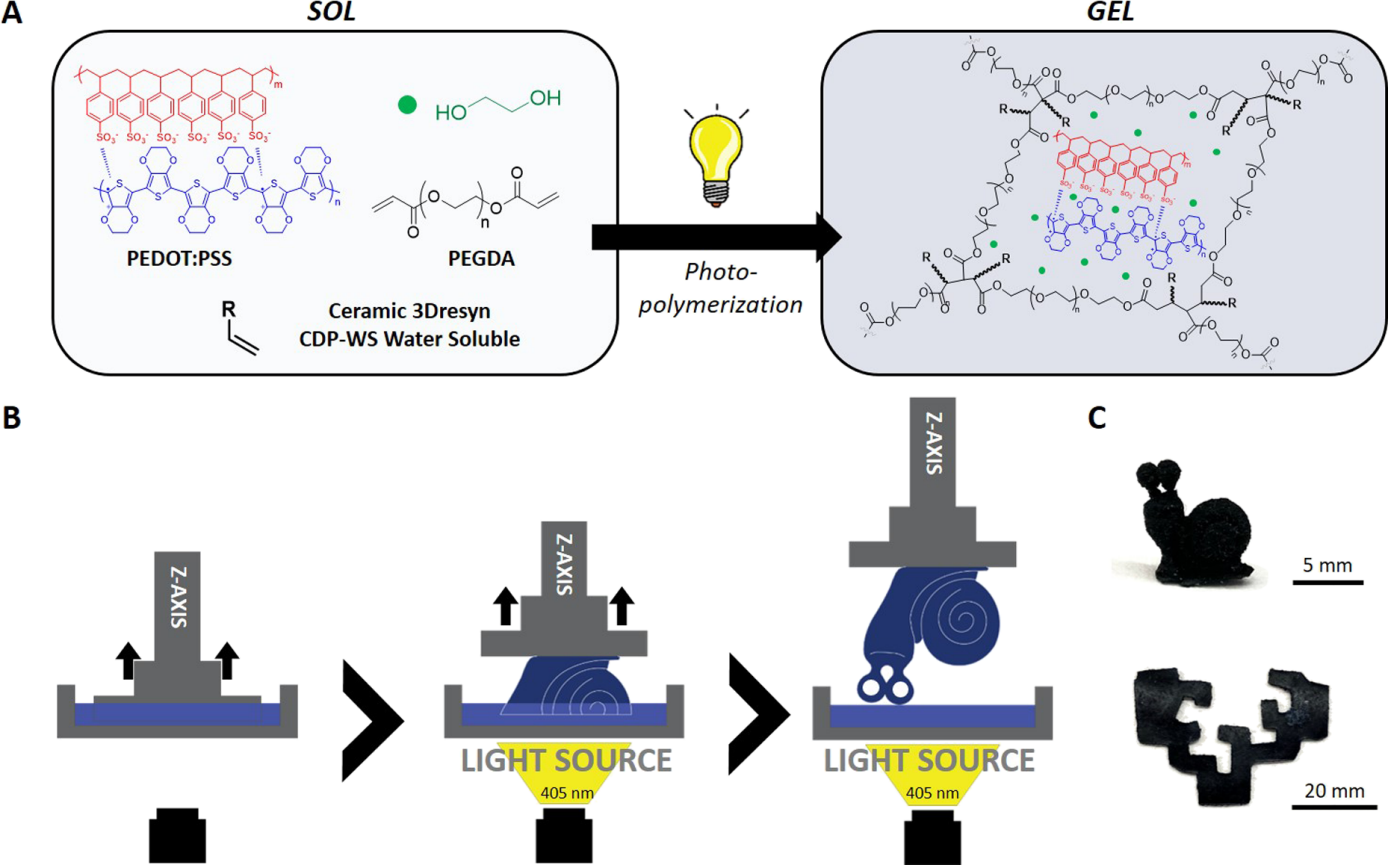
(A) Synthesis of Conductive Hydrogels by the Photopolymerization Reaction, (B) Fabrication of Shape-Defined 3D Hydrogels by DLP of the Photopolymerizable Inks and C) Pictures of the 3D Printed Pieces

Therefore, herein we present the design of photocurable and conductive polymer inks to be processed by DLP leading to shape-defined conductive hydrogels for biosensing. The polymer ink is obtained through a precise formulation based on the dispersion of PEDOT:PSS in a photocurable matrix composed of poly(ethylene glycol) diacrylate (PEGDA), an aqueous commercial resin (vinyl monomers) that favors the dispersion of PEDOT in the PEGDA matrix and enhances its conductivity in conjunction with ethylene glycol (EG) and a photoinitiator (Scheme 1). All the components were commercially available, and the 3D printable ink could be easily prepared by simple mixing without any treatment or evaporation step. The influence of the number-average molecular weight of PEGDA (*M*_n_ = 250, 575, and 700 Da) on the photopolymerization kinetics and printing resolution of the inks, as well as on the swelling ratio and mechanical properties of the printed hydrogels, is studied. Moreover, the electrical conductivity of the printed materials is measured. Finally, their application as long-term bioelectrodes in electrocardiography (ECG) and electromyography (EMG) is investigated and compared with the recording properties of commercial Ag/AgCl medical electrodes.

## 2 Materials and Methods

### 2.1 Materials

Clevios PH 1000 PEDOT:PSS (1.3 wt %) aqueous solution was purchased from Heraeus. Ceramic 3Dresyn CDP-WS Water-Soluble and Fine Turner FT2 ultrafast photoinitiator were supplied by 3Dresyns. Poly(ethylene glycol) diacrylate (PEGDA, *M*_n_ = 250, 575, and 700 Da) and ethylene glycol (EG) were provided by Sigma-Aldrich.

### 2.2 Photopolymerizable and Conductive Hydrogels

#### 2.2.1 Photopolymerizable Conductive Ink

The photocurable conductive ink was prepared by mixing PEDOT:PSS aqueous solution (50 wt %) with 3Dresyn CDP-WS (34 wt %), Fine Turner FT2 (4 wt %), EG (4 wt %), and PEGDA (8 wt %) of different *M*_n_ (250, 575, and 700 Da). Samples were named as PEDOT_*x*__PEGDA_*n*_ where *x* refers to PEDOT percentage and *n* to the *M*_n_ of PEGDA. The mixture was protected with aluminum paper from light and stirred for 24 h.

#### 2.2.2 Photopolymerization Reaction Kinetics

The photopolymerization reaction conditions were studied by Fourier transform infrared spectroscopy (FTIR) and photorheology.

FTIR spectra were recorded in the attenuated total reflectance (ATR) mode in a Thermo scientific model Nicolet 6700 FTIR spectrometer, with a resolution of 8 cm^–1^, mirror speed of 0.3165, and 1 scan. Samples were placed in a zinc selenide glass, and ATR-FTIR spectra were recorded at room temperature every 1.16 s by exposing the photocurable inks to UV light (wavelength = 365 nm, power = 2 mW cm^–2^) for 30 s. The conversion was calculated with eq 1 by measuring the area of the peaks located at 953 and 977 cm^–1^, which correspond to the C=C out of plane bending vibrations:
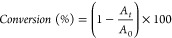
1where *A*_*t*_ is the area of the band at a time *t* and *A*_0_ is the area of the band at zero time.

Photorheological measurements were carried out at room temperature in an AR-G2 rheometer (TA Instruments) using a UV-light lamp (wavelength = 365 nm, power = 2 mW cm^–2^), oscillation stress of 100 Pa, and 0.1 Hz frequency. The gel point was determined by placing the samples on a glass parallel plate of 20 mm diameter, letting them stabilize for 60 s to be subsequently irradiated for 5 s, and continuing to register the elastic modulus (*G*′) and loss modulus (*G*′′) until a plateau was reached.

### 2.3 Digital Light 3D Printing (DLP) of Conductive Hydrogels

The photopolymerizable conductive inks were placed into the vat of the 3D printer (Phrozen Sonic Mini) and exposed to UV light (wavelength = 405 nm, power = 2 mW cm^–2^). Printing patterns were first designed with Autodesk Inventor 2021 software and then printed at different irradiation times (2, 5, and 10 s) and layer heights (0.05, 0.1, and 0.2 mm) and a lifting speed of 50 mm min^–1^.^41^

### 2.4 Characterization of Printed Hydrogels

#### 2.4.1 Scanning Electron Microscopy (SEM)

Measurements were performed on a Hitachi Tabletop Microscope (TM3030 series) at a 15 kV force field, running in a point-by-point scanning mode. The samples were placed on an aluminum holder with double-sided carbon tape and introduced into the SEM chamber. Finally, SEM images were analyzed with the software ImageJ.

#### 2.4.2 Swelling Assays

Discs of 1 mm thickness and 1 cm diameter were printed and dried at 25 °C. Subsequently, the dry discs were weighted (*W*_*0*_) and immersed in Milli-Q water at 25 °C. At established times, the samples were removed from Milli-Q water, externally dried with filter paper to eliminate the excess water that could remain on the surface, and weighed (*W*_*t*_). The swelling percentage (*S_w_*) was calculated according to eq 2:
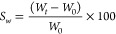
2

Besides, the gained volume in the swelling process was also calculated by measuring the dimensional changes of diameter and thickness of the discs at each established time. Samples were analyzed in triplicate and results were expressed as mean ± standard deviation.

#### 2.4.3 Electrical conductivity

Discs of 10 mm diameter and 0.5 mm thickness were printed and their electrical conductivity was measured in dry and swollen states using a four-point probe Ossila Sheet. The swelling was carried out in Milli-Q water at 25 °C, and the drying step at 25 °C and room atmosphere, or 85 °C in an oven. The electrical conductivity was calculated taking into account the thickness of each sample at each condition tested. Samples were analyzed in triplicate and testing three different sample areas and results were expressed as mean ± standard deviation.

#### 2.4.4 Electrochemical characterization

Cyclic voltammetry was performed using a VMP-3 potentiostat (Biologic Science Instruments) in a three-electrode setup, employing a platinum wire as the counter electrode, Ag/AgCl as the reference electrode, and glassy carbon as the working electrode. Hydrogels were fabricated by depositing 5 μL of the ink onto the glassy carbon electrode, followed by a photopolymerization step with UV light irradiation. Finally, they were cleaned in water for 5 min. 0.1 M NaCl aqueous solution was used as the electrolyte, and it was purged with nitrogen for 15 min before the experiment. Cyclic voltammetry measurements were carried out in the potential range of −0.2 to 0.5 V vs. Ag/AgCl at 20 mV s^–1^ and 20, 50, 100, 150, and 200 mV s^–1^ for the scan rate experiment.

#### 2.4.5 Tensile Test

Type V probes were printed. The tensile test was applied to probes in the dried and swollen state by the ASTM D638 criterion universal testing system using an Instron 5569 in a single uniaxial tensile mode (n = 5). The controlled tension force (load cell of 100 N) was applied at a rate of 1 mm min^–1^, and the results were plotted as stress–strain curves. Samples were analyzed in triplicate and results were expressed as mean ± standard deviation.

### 2.5 Electrocardiography (ECG) and Electromyography (EMG)

#### 2.5.1 Electrodes fabrication

A Kapton sheet of 75 μm thickness was covered with 10 nm of Ti and 200 nm of gold by thermal evaporation (Alliance Concept). The coated Kapton was laser cut (LPKF protolaser S) with the desired shape (1 cm diameter and 2 × 0.2 cm connection for ECG recordings, 4 × 4 cm and 1 × 0.5 cm connection for EMG recordings). Subsequently, samples of these shapes were printed, swollen in Milli-Q water, and attached to the Au electrode with a tape, resulting in PEDOT-based electrodes. In all cases, hydrogels were used in their wet state to improve the adhesion between the hydrogel and the skin, and the hydrogel and the gold.

#### 2.5.2 ECG acquisition and signal processing

The ECGs were recorded using a Sienna Ultimate device plugging the two PEDOT-based electrodes to compare to ECG entries. The reference electrode (RE) used was always a fresh medical electrode (Gima 33 371 universal electrodes for ECG, diameter 48 × 50 mm) located on the left bottom of the chest. The working electrode (WE) and counting electrode (CE) were located on the wrists. The signal was recorded with a hardware notch filter at 50 Hz. Before plotting the different ECGs, the signal was processed using an infinite impulse response (IIR) notch filter at 50, 100, and 150 Hz. The electrodes were stored between measurements and reused for the long-term recordings at different times.

#### 2.5.3 EMG recording and stimulation experiments

Muscle stimulation was applied on the quadriceps using a Boston Tech WE-122 Electrotherapy Unit equipment, employing a program that sends 380 μs pulses at a frequency of 104 Hz. When a current of 10 mA was applied (visual muscle contraction was observed), the muscle activity was recorded using PEDOT-based electrodes. For contraction/relaxation tests, EMGs were recorded with a Sienna Ultimate device plugging the bottom electrode to the reference using PEDOT electrodes when a healthy volunteer contracted and relaxed the quadriceps. In all cases, signals were recorded with a hardware notch filter, and the plot signals were filtered with EDFViewer using notch Filters at 50, 100, and 150 Hz and a high-pass filter at 10 Hz of order 8. The electrodes were stored between measurements and reused for the long-term recordings at different times.

## 3 Results and Discussion

### 3.1 Development of photopolymerizable PEDOT inks

**Figure 1:**
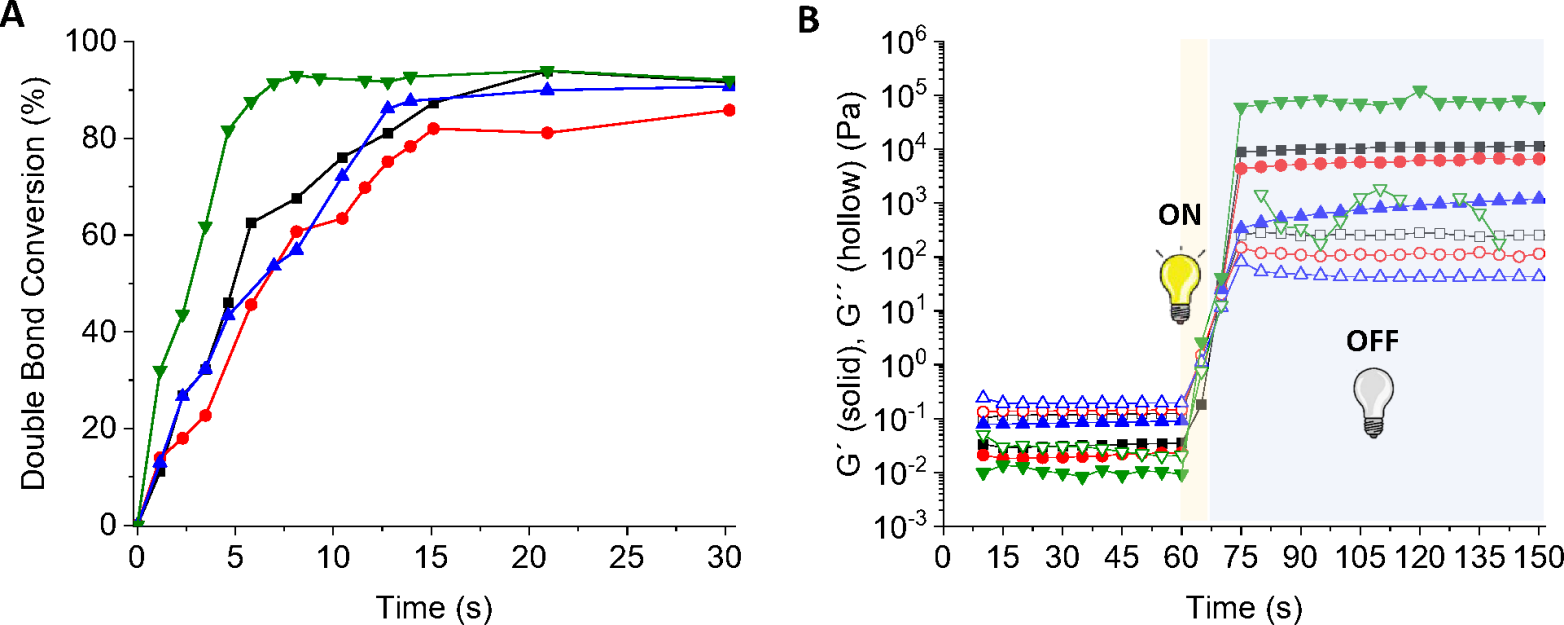
(A) Evolution of the conversion during the photopolymerization process of the inks PEDOT_0.00__PEGDA_700_ (green triangles), PEDOT_0.65__PEGDA_250_ (black squares), PEDOT_0.65__PEGDA_575_ (red circles), and PEDOT_0.65__PEGDA_700_ (blue triangles). (B) Rheological measurements of the elastic modulus (*G*′) and loss modulus (*G*′′) to determine the gel point when the inks are irradiated for 5 s (in the interval from 60 to 65 s).

A schematic representation of the overall fabrication process of the conductive hydrogel is shown in Scheme 1. First, the PEDOT:PSS aqueous solution that provides electrical properties to the final material was dispersed in the photocurable matrix composed of 3Dresyn CDP-WS (vinyl monomers), PEGDA as a cross-linking agent, and the photoinitiator Fine Turner FT2. The PEDOT percentage was optimized to obtain homogeneous inks and maximize the electrical conductivity. In this regard, 0.65% PEDOT was the optimal quantity that could be homogeneously dispersed within the photopolymerizable matrix without hindering the digital light printing (DLP) process, as higher quantities of PEDOT could avoid the light penetration during DLP leading to defects in the printed objects. To increase the conductivity of the final material, a little quantity of EG was added to the ink formulation.^42,43^ It is well-known that the PEDOT:PSS conductivity can be enhanced by a solvent doping treatment, and ethylene glycol (EG) is commonly used for such purposes. EG screens the ionic interaction between PEDOT and PSS by forming hydrogen bonds with PSS, which results in a strong step separation between PEDOT and PSS, allowing the linear orientation of the PEDOT chains.^44,45^ The incorporation of EG in the inks gave rise to a 3-fold increase of the conductivity, from 2.5 × 10^–3^ S cm^–1^ to 7.1 × 10^–3^ S cm^–1^ for PEDOT_0.65__PEGDA_700_. It is noteworthy that the energy transfer process is also influenced by thermal conductivity.^46^ In this regard, the employment of EG to align the PEDOT chains within the hydrogel network reduces the structural disorder, and more phonon-like modes are available to contribute to heat conduction.^47,48^ Then, hydrogels were formed by photopolymerization of the previous photocurable mixture (PEDOT:PSS, PEGDA, vinyl resin, EG, and FT2) using UV light, leading to a cross-linked network where PEDOT is trapped. The photopolymerization kinetics were studied by ATR-FTIR (Figure S1). The peaks located at 953 and 977 cm^–1^, which correspond to the C=C out-of-plane bending vibrations, disappear as the photopolymerization reaction proceeds. The conversion was calculated using eq 1 by measuring the area of these peaks over time (Figure 1A). Results show a fast conversion of PEDOT_0.00__PEGDA_700_ reaching a plateau in 5 s. The kinetics were slightly decreased when PEDOT is incorporated, due to the blue color of PEDOT that limits the light penetration whereas the control sample without PEDOT is totally transparent. However, no significant differences were observed for inks prepared with different molecular weights of PEGDA. The fast photocuring kinetics make those inks ideal candidates for DLP 3D printing. One important parameter for DLP of photopolymerizable hydrogels is their ability to rapidly reach the gel point, the transition from liquid to solid state, during printing. It is known that the free-radical photopolymerization normally reaches the gel point at 40–60% conversion.^49^ This conversion is achieved at approximately 5 s for PEDOT-containing samples. Therefore, the gel point was determined by photorheology, applying a 5 s UV light irradiation (Figure 1B), and the results show that all inks reach the gel point in less than 5 s. This demonstrates the capability of the inks to achieve the gel point without being necessary to reach a full conversion, allowing researchers to obtain 3D architectures by DLP in very short times.

### 3.2 Digital Light Printing of PEDOT Conductive Hydrogels

**Figure 2:**
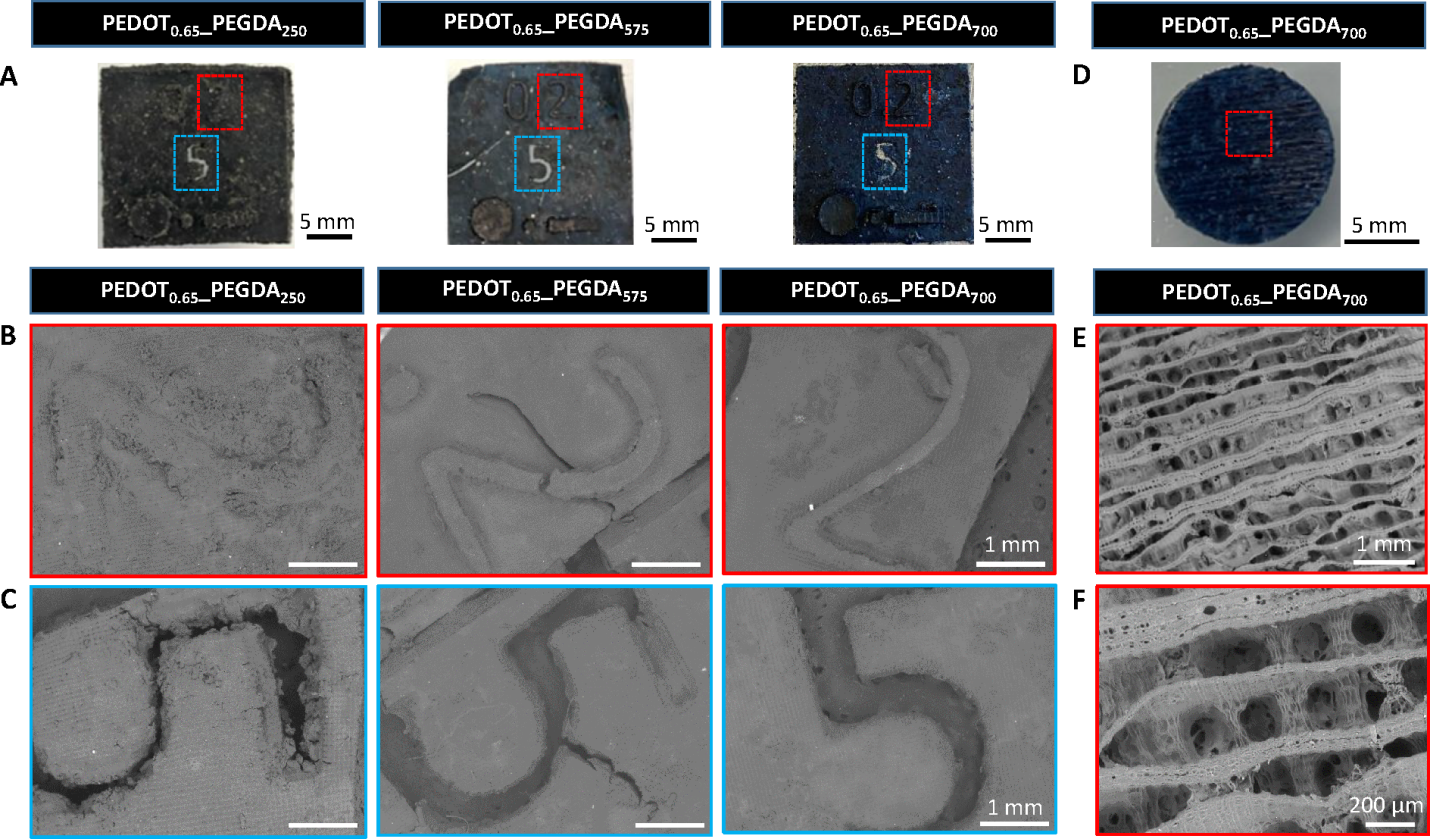
(A) Pictures of hydrogel pieces printed by DLP (0.2 mm layer height, 5 s laser irradiation). SEM images of different areas of the previously printed pieces showing (B) reliefs (dashed red areas in part A) and (C) holes (dashed blue areas in part A). Scale bar = 1 mm. (D) Picture of a porous PEDOT_0.65__PEGDA_700_ scaffold printed by DLP (0.2 mm layer height, 5 s laser irradiation). (E) SEM image of a representative area of the previous porous scaffold (dashed red area in part D) and (F) zoom-in to visualize the porous structure and pore dimensions.

Digital light printing (DLP) was used to pattern conductive hydrogels with predesigned architectures during the photopolymerization process, leading to three-dimensional shape-defined network structures (Scheme 1B–C). Optimal printing conditions were investigated by DLP of 3D patterns with reliefs and holes at different irradiation times (2, 5, and 10 s) and layer heights (0.05, 0.1, and 0.2 mm) using the photopolymerizable ink PEDOT_0.65__PEGDA_250_ (Figure S2A and B). When low irradiation times (2 s) were employed, the printing patterns did not show the designed structure because the ink could not photocure successfully, as proven by the double bond low conversion (<30%) (Figure 1A). Increasing the exposure time to UV light up to 5 s gave rise to patterns with better printing resolution. However, an excess of photopolymerization time, while printing successfully the reliefs of the pattern, did not allow printing the hollow parts of the designed piece that were fully covered by the ink during DLP. A high cross-link density makes printed hydrogels more rigid and brittle, giving rise to totally deformed objects after printing or to the appearance of scratches, which is even more evident at lower layer thicknesses (Figure S2B–D). Therefore, 5 s of light exposure was chosen as the optimal time that affords accurate photopolymerization rates, with over 50% double bond conversion (Figure 1A), ensuring high printing resolution. Then, the printing resolution was fit by varying the layer height. It was observed that lower layer heights (0.05 and 0.1 mm) did not allow researchers to obtain shape fidelity patterns, whereas using a layer height of 0.2 mm gave rise to a shape fidelity pattern that exhibited all the features of the model piece, i.e., reliefs and holes, as was corroborated by SEM (Figure S2E and F). When the system absorbs too much light, the use of thin layer heights results in not very good resolution because the photopolymerization process is deflected to unspecified points of the printing pattern, causing imperfections in the final material. However, when larger layer heights of the material absorb the same amount of light, this deviation is corrected and the reaction occurs only at the locations specified in the printing pattern. Once the printing parameters were optimized, the influence of the molecular weight of PEGDA on the printing fidelity was studied (Figure 2A–C).

Patterns printed with the inks formulated with high molecular weight PEGDA, PEDOT_0.65__PEGDA_575,_ and PEDOT_0.65__PEGDA_700_ show better printing resolution and shape fidelity than those of PEDOT_0.65__PEGDA_250_. One might think this behavior depends on the acrylate functional group concentration, which in turn depends on the length of the PEGDA chains. Nevertheless, in general, all the samples have the same concentration of acrylate groups, as the PEGDA quantity in the ink formulation is only 8%wt. Therefore, this behavior seems to be more closely correlated with the PEGDA molecular weight than the acrylate concentration, which has not been fully explained in the literature yet.^50^ Consequently, the PEGDA chain length affects the printability resolution, with PEDOT_0.65__PEGDA_700_ hydrogels becoming ideal candidates for processing by DLP. To illustrate this, different complex 3D structures, i.e., a snail and the UPV/EHU logo, were successfully printed using the selected ink, PEDOT_0.65__PEGDA_700_ (Scheme 1C). Apart from that, the possibility to print high-resolution microporous hydrogel scaffolds with PEDOT_0.65__PEGDA_700_ was tested (Figure 2D). Morphological analysis by SEM showed a layer-by-layer microporous structure with pore diameters of ∼130 μm (Figure 2E and F).

### 3.3 Characterization of the Printed PEDOT Conductive Hydrogel

**Figure 3:**
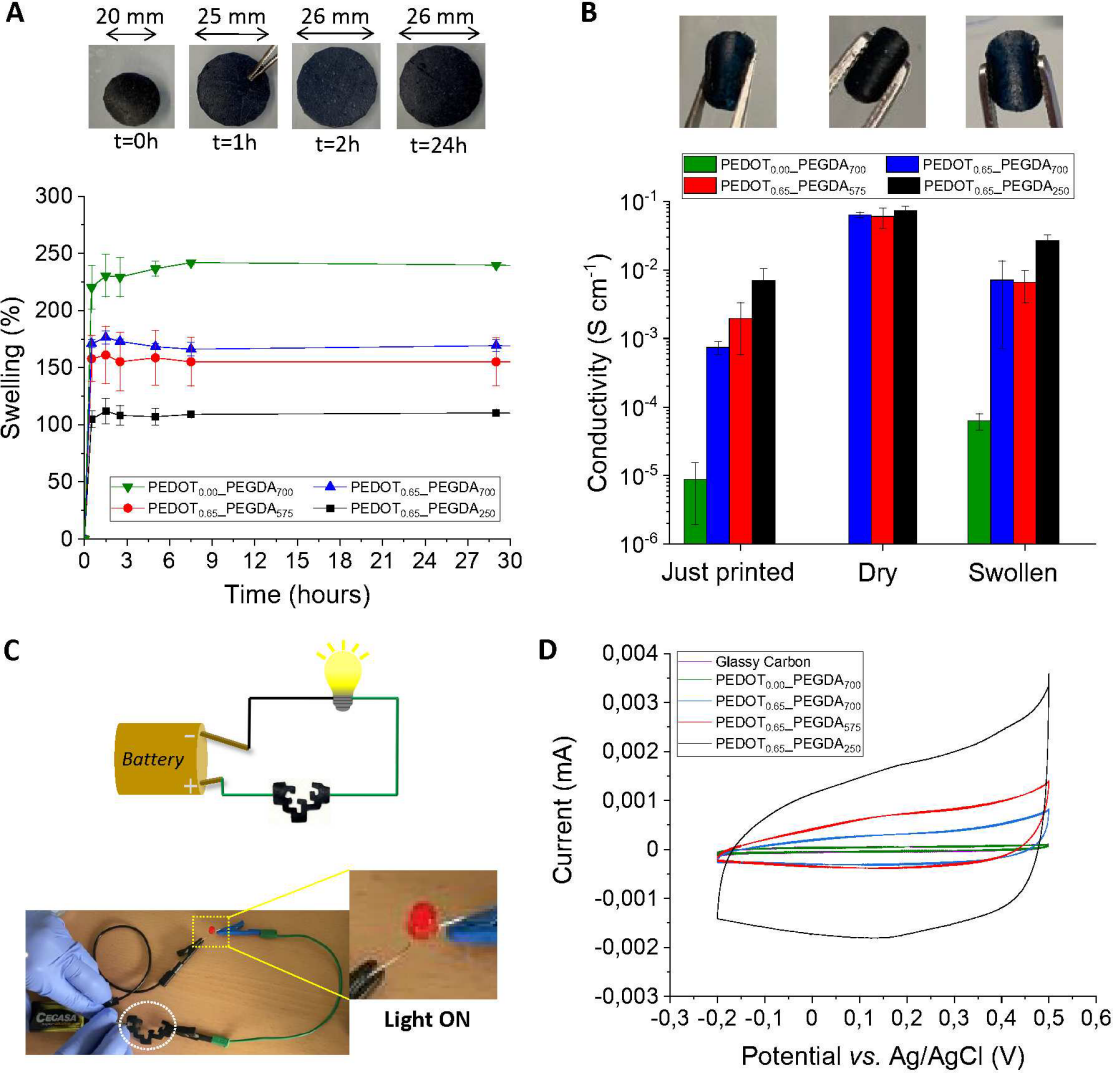
(A) Photographs of the hydrogel PEDOT_0.65__PEGDA_575_ just printed (*t* = 0 h) and during its swelling in water (top). Swelling curves of the printed hydrogels: PEDOT_0.00__PEGDA_700_ (green triangles), PEDOT_0.65__PEGDA_250_ (black squares), PEDOT_0.65__PEGDA_575_ (red circles), and PEDOT_0.65__PEGDA_700_ (blue triangles) in water (bottom). (B) Photographs of the printed hydrogels and electrical conductivity of PEDOT_0.00__PEGDA_700_ (green bars), PEDOT_0.65__PEGDA_250_ (black bars), PEDOT_0.65__PEGDA_575_ (red bars), and PEDOT_0.65__PEGDA_700_ (blue bars). (C) Lab-made electrical circuit to conduct the electrical current from the battery to the light bulb, passing through the printed UPV/EHU logo with a PEDOT_0.65__PEGDA_700_ hydrogel, as a visual proof-of-concept of the hydrogel conductivity. The dashed white circle highlights the printed logo, and the dashed yellow rectangle, the localization of the light bulb switched on. (D) Cyclic voltammograms of the 3D printed hydrogels, PEDOT_0.00__PEGDA_700_ (green curve), PEDOT_0.65__PEGDA_250_ (black curve), PEDOT_0.65__PEGDA_575_ (red curve), and PEDOT_0.65__PEGDA_700_ (blue curve), over a glassy carbon electrode (purple curve) in 0.1 M NaCl aqueous solution at 20 mV s^–1^.

An important feature of the hydrogel scaffolds to be employed for bioelectronics is their ability to retain their shape in the swollen state.^51^ The printed patterns showed a fast swelling rate (S_w_) during the first hour, reaching a plateau within 2 h and retaining its structure without breaking (Figure 3A). Hydrogels, which are formed by free-radical-mediated cross-linking at the PEGDA chain ends creating a covalent network, are able to hold water in the cross-linked matrix due to the presence of hydrophilic PEGDA and the resin backbone, which form hydrogen bonds with water, at the same time that the cross-linking points of the polymer network prevent its disaggregation. Thus, the mesh size of the hydrogels decreases with the decrease of the PEGDA chain length, which indeed increases the cross-link density and decreases their swelling ability.^52,53^ The presence of PEDOT in the ink formulations decreases the cross-linking density and increases the hydrophobicity, which results in a lower swelling from S_w_ = 230 ± 19% for PEDOT_0.00__PEGDA_700_ to S_w_ = 176 ± 6% for PEDOT_0.65__PEGDA_700_. Among samples containing PEDOT, hydrogels formed by shorter PEGDA chains, e.g., PEDOT_0.65__PEGDA_250_, possess a higher cross-link density than those formed by longer PEGDA chains, e.g., PEDOT_0.65__PEGDA_575_ and PEDOT_0.65__PEGDA_700_. Therefore, PEDOT_0.65__PEGDA_250_ hydrogels possess the lowest swelling ability (S_w_ = 112 ± 11%), as shown in Figure 3A. Besides, it was also proven that the printed hydrogels were swollen homogeneously in the three dimensions, reaching a maximum swelling state at around 38% diameter and thickness (Figure S3).

As the presence of water clearly influences the conductive properties of the printed materials, Figure 3B shows the electrical conductivity values of the synthesized hydrogels in the dry and swollen states. Immediately after printing, PEDOT-containing samples show conductivity values of 10^–2^ S cm^–1^ for PEDOT_0.65__PEGDA_250_ and 10^–3^ S cm^–1^ for PEDOT_0.65__PEGDA_575_ and PEDOT_0.65__PEGDA_700_. This happens because the PEDOT_0.65__PEGDA_250_ hydrogel absorbs less water than the other two gels (Figure 3A), and hence, PEDOT molecules are more concentrated and ordered in PEDOT_0.65__PEGDA_250_.

After drying at 25 °C, the hydrogel scaffolds increase their electronic conductivity until 10^–1^ S cm^–1^ in all cases, as there is no water in the structure, leading to a better reorganization of the PEDOT chains. In this state, PEDOT acts as a very good electrical conductor. The annealing of the hydrogels at 85 °C did not produce any significant increase in the conductivity as compared with hydrogels dried at 25 °C (Figure S4). When samples are swollen after drying, their conductivity decreases 1 order of magnitude in comparison with their dry state but remains higher than those just after printing, while they retain their flexibility when they are stretched with tweezers as shown in the photographs. It is worth noting that the PEDOT_0.00__PEGDA_700_ sample used as control, without PEDOT in its structure, only shows low conductivity values in its swollen state due to the ionic conductivity and it does not show electrical conductivity in its dry state, as expected, proving the key role of PEDOT within the ink formulation for conductive purposes. As a visual proof-of-concept of hydrogel conductivity, a lab-made electrical circuit that incorporates the printed hydrogel shaping the UPV/EHU logo was built (Figure 3C). When the battery is connected, the printed scaffold is able to transfer the electrical current to the LED switching it on (see Video S1). The electroactive behavior of our hydrogels has also been tested by cyclic voltammetry in 0.1 M NaCl aqueous solution (Figure 3D and Figure S5). The cyclic voltammograms (CV) of the hydrogels containing PEDOT show a capacitive behavior, with a broad anodic peak at 0.4 V and a broad cathodic peak at 0.15 V, not observed in the case of the hydrogel without PEDOT. The electroactivity of the hydrogels follows the same tendency as the conductivity measurements of the hydrogels after printing (Figure 3B), being higher in the case of hydrogels formed with a lower molecular weight of PEGDA. Moreover, the CVs of PEDOT_0.65__PEGDA_700_ at different scan rates show a proportional increase of the anodic and cathodic currents with the scan rate, which means that the redox process is not limited by diffusion and the whole hydrogel is involved in the electrochemical process (Figure S5).

**Figure 4:**
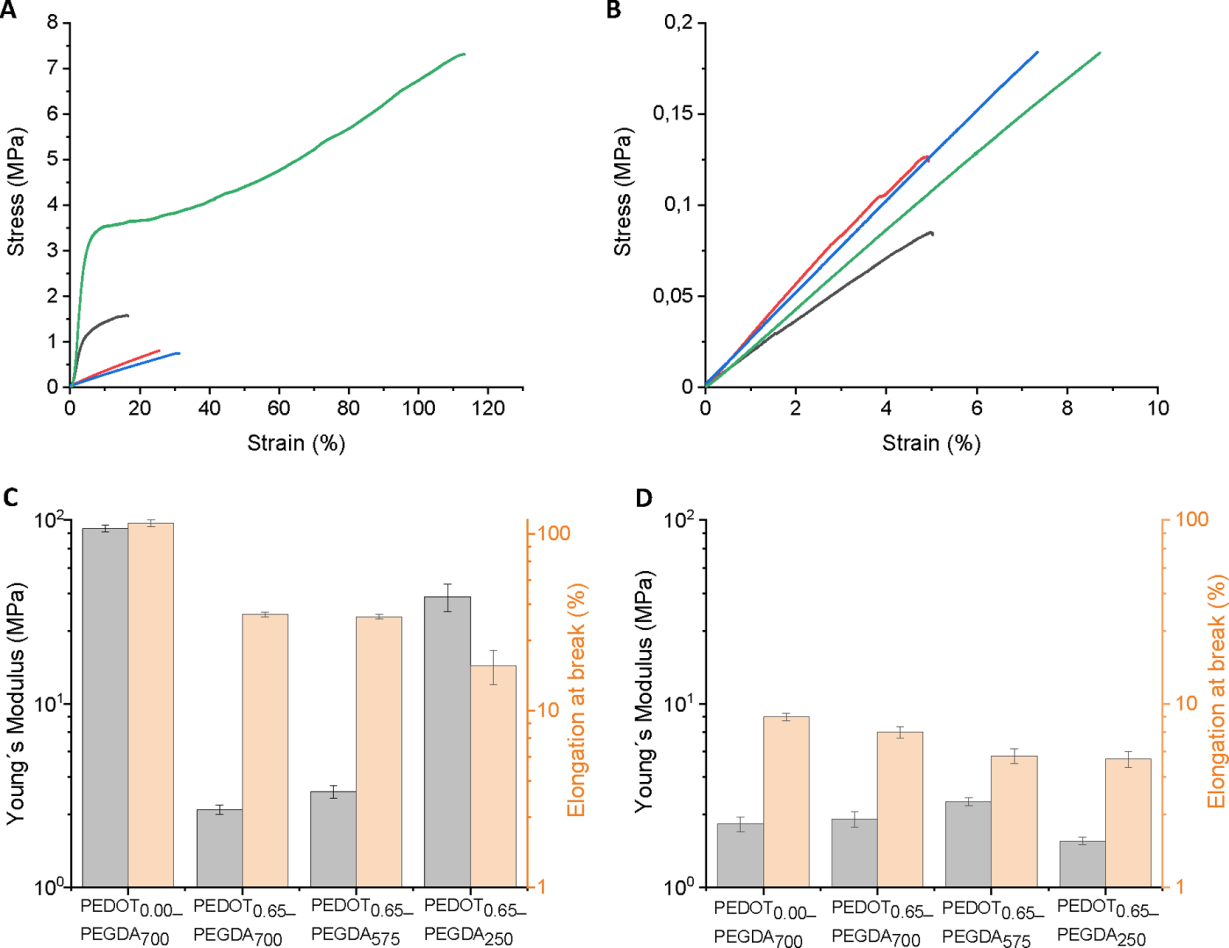
Stress–strain curves of type V probes’ printed hydrogels: PEDOT_0.00__PEGDA_700_ (green curve), PEDOT_0.65__PEGDA_250_ (black curve), PEDOT_0.65__PEGDA_575_ (red curve), and PEDOT_0.65__PEGDA_700_ (blue curve) in the (A) dry and (B) wet states. The Young’s modulus and elongation at break were obtained from the strain–stress curves of gels in the (C) dry and (D) wet states.

The mechanical properties of printed hydrogels in the form of type V probes were determined by measuring the Young’s modulus and elongation at break in the dried and swollen states (Figure 4A–B). In the dried state, the control hydrogel PEDOT_0.00__PEGDA_700_ possesses the highest Young’s modulus , 90.2 ± 3.9 MPa, and elongation at break, 115% (Figure 4C), due to its high photopolymerization reaction conversion previously discussed. The presence of PEDOT within the hydrogel formulation, PEDOT_0.65__PEGDA_700_, decreases the Young’s modulus up to 2.7 ± 0.2 MPa as well as the elongation at break (35%). This can be attributed to the fact that the black color of the PEDOT dispersion hinders the light penetration through the ink during the photopolymerization process, decreasing the cross-linking degree between the chains of PEGDA and the resin. In addition to this, it was observed that the Young’s modulus increases as the molecular weight of PEGDA decreases, reaching values of 38.3 ± 6.6 MPa for PEDOT_0.65__PEGDA_250_, whereas the elongation at break decreases up to 18%. As above-mentioned, hydrogels formed by shorter PEGDA chains, e.g., PEDOT_0.65__PEGDA_250_, possess a higher cross-link density than those formed by longer PEGDA chains, e.g., PEDOT_0.65__PEGDA_575_ and PEDOT_0.65__PEGDA_700_, exhibiting a lower swelling ability (Figure 3A). As the cross-link density increases, hydrogels become stiffer, possessing a higher Young’s modulus, and more brittle, exhibiting a lower elongation at break, as shown in Figure 4C. Conversely, in the swollen state, all hydrogels have lower and similar stiffness values, in the range of 2–3 MPa (Figure 4D), due to the hydrated state and the water held within their network. In terms of mechanical properties, these results are comparable to the Young’s modulus of the forearm skin, 1.03 ± 0.06 MPa, where electrodes are placed for ECG recordings.^54^ Therefore, taking into account that the conductivity and mechanical properties of the printed conductive hydrogels in their wet state are not highly influenced by the hydrogel formulation and that PEDOT_0.65__PEGDA_700_ hydrogels possess the highest printing fidelity for the fabrication of shape-defined electrodes, those are selected for further bioelectronics tests.

### 3.4 Testing of 3D Printed PEDOT Hydrogels in Electrocardiography (ECG) and Electromyography (EMG) Recordings

**Figure 5:**
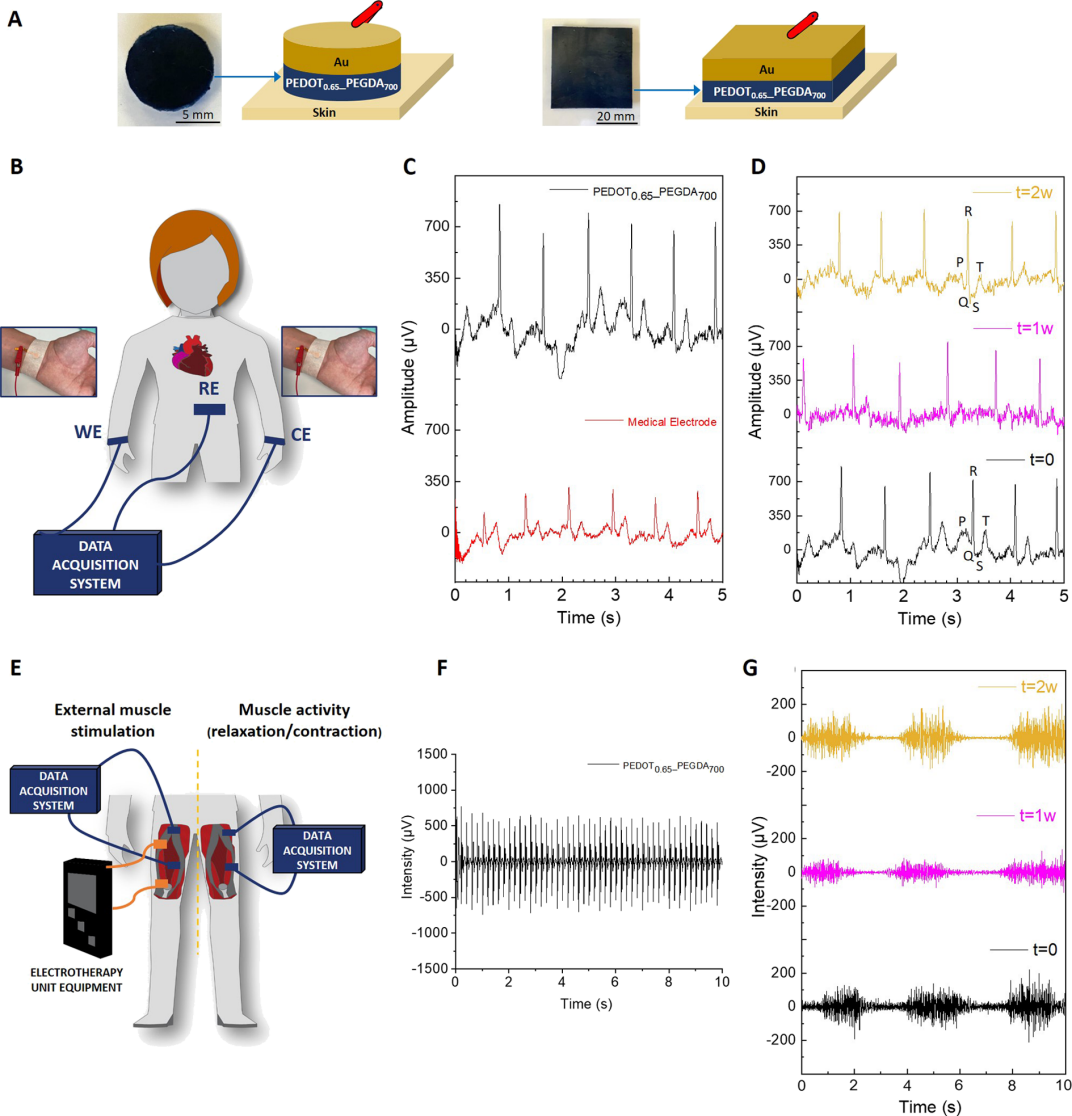
(A) Pictures of the digital light 3D printed PEDOT_0.65__PEGDA_700_ hydrogels, with a round shape for ECG and square shape for EMG, and schematic representation of the built-up electrodes. (B) Scheme of the ECG performed. (C) Comparison of ECG signals using a medical electrode and synthesized PEDOT_0.65__PEGDA_700_ hydrogel. (D) ECG signals recorded with PEDOT-based hydrogel over time (*t* = 0, 1, and 2 weeks (w)). (E) Scheme of external muscle electrostimulation and muscle activity recording experiments. (F) EMG response of PEDOT_0.65__PEGDA_700_ electrode after electrostimulation of a female volunteer. (G) Evolution over time (*t* = 0, 1, and 2 weeks (w)) of the EMG signals generated by contraction/relaxation of the quadriceps.

The 3D printed conductive hydrogels (Figure 5A and Table S1) were tested as cutaneous electrodes for health monitoring on a healthy volunteer. First, they were employed for electrocardiogram (ECG) recording, a medical test used in preventive medicine to detect heart problems by measuring the electrical activity generated by the heart contraction. For such purpose, the PEDOT_0.65__PEGDA_700_ hydrogel in the wet state was used as working electrode (WE) and counter electrode (CE) for the ECG recordings, whereas an Ag/AgCl medical electrode was employed as reference electrode (RE) (Figure 5B). The results show that the electrocardiogram (ECG) recorded when PEDOT_0.65__PEGDA_700_ hydrogel is used (black curve) displays more intense signals than the standard medical electrode (red curve) (Figure 5C) as well as a good detection of PQRST waveforms, physiological signals that provide valuable information for diagnosis and rehabilitation (Figure 5D). The signal-to-noise ratio of the electrodes has also been determined (Figure S6). The plot contains the extracted noise from the raw data with notch filters at 50, 100, and 150 Hz as well as a low-pass filter at 150 Hz and a high-pass filter at 0.05 Hz, as recommended by the American Heart Association. As can be observed, the signal-to-noise ratio of the electrodes built up with our hydrogels is much lower than this one of the standard medical electrodes, at the same time that the QSR signal is much higher with our electrodes.^55^ Besides, the PEDOT_0.65__PEGDA_700_ bioelectrode showed long-term stability by reproducing the high-quality ECG signals after 2 weeks, which is indicative of the material stability over time. In this regard, the stability was corroborated by the degradation assay carried out by weight loss and SEM (Figure S7). The printed hydrogels in a wet state kept their initial mass for up to 2 weeks and did not experience significant changes in the surface morphology during this period.

Apart from that, the printed PEDOT_0.65__PEGDA_700_ hydrogel was further tested for electromyography (EMG) recording, a measurement of the electrical activity produced by skeletal muscles (Figure 5E). Two different experiments were carried out using the PEDOT_0.65__PEGDA_700_ hydrogel as WE: EMG recording when an external electrostimulation is applied to the quadriceps and muscle activity when the quadriceps are relaxed and contracted. For external electrostimulation, a current of 10 mA was applied (380 μs pulses, frequency = 104 Hz) to the quadriceps of a healthy volunteer, and the muscle activity was recorded (Figure 5F). It can be clearly observed when the muscle is being stimulated with very good signal quality and intensity. On the other hand, the PEDOT_0.65__PEGDA_700_ hydrogel was used to monitor muscle contraction/relaxation when a healthy volunteer contracted and relaxed their quadriceps in alternating 2 s cycles (Figure 5G). The results demonstrate that the developed bioelectrode can monitor the muscle contraction and relaxation steps with accurate intensity signals. Furthermore, the bioelectrode also exhibits a long-term activity, showing reproducible signals for at least 2 weeks. Overall, these results reveal that the printed conductive hydrogels show a long-term activity and stability to be used as electrodes for physiological recording and health monitoring.

## 4 Conclusion

Photopolymerizable and conductive PEDOT-based inks have been formulated to be employed for additive manufacturing of 3D scaffolds by DLP. All the components of the ink were commercially available, and the 3D printable ink could be easily prepared by simple mixing without any treatment or evaporation step. The photopolymerization reaction was very fast, achieving the gel point in less than 5 s, which makes the designed inks ideal candidates to be processed using commercial 3D printers. The molecular weight of the PEGDA employed within the ink formulation empirically affected the resolution printability, with the PEDOT_0.65__PEGDA_700_ hydrogel showing the highest printing fidelity. Moreover, the capacity of the printed hydrogels to hold water confers them soft mechanical properties, with Young’s moduli in the order of 3 MPa, which makes them ideal candidates for tissue engineering applications. In addition to this, the printed hydrogels exhibited high electrical conductivity, between 10^–1^ and 10^–2^ S cm^–1^, enabling them to be employed as bioelectrodes for long-term ECG and EMG recordings with enhanced detection signals compared to commercial Ag/AgCl medical electrodes. Overall, these findings suggest the developed 3D printed hydrogels present great potential as flexible wearable electronic devices for a variety of biological and engineering applications.

## Supporting Information Available

Video of the lab-made electrical circuit while running (MP4)
ATR-FTIR spectra during the photopolymerization process; pictures and SEM images for printing process optimization; evolution of the diameter and thickness of hydrogels during swelling; mechanical properties of hydrogels in the dry and wet states; degradation assay (PDF)
