# Supplementary material for: Digital Light 3D Printing of PEDOT-Based Photopolymerizable Inks for Biosensing
Source: ACS Appl Polym Mater. 2022 Aug 10;4(9):6749–59. doi: 10.1021/acsapm.2c01170 (PMC9469088; doi:10.1021/acsapm.2c01170)
Supplement: Supplementary file 2 — ap2c01170_si_002.pdf [file ap2c01170_si_002.pdf]

SUPPORTING INFORMATION

Digital Light 3D Printing of PEDOT-based Photopolymerizable Inks for Biosensing

Naroa Lopez-Larrea,[1] Miryam Criado-Gonzalez,[1] Antonio Dominguez-Alfaro,[1] Nuria Alegret,[2,3] Isabel del Agua,[4] Bastien Marchiori,[4] and David Mecerreyes[1,5]**

[1] POLYMAT, University of the Basque Country UPV/EHU, Paseo Manuel de Lardizabal 3, 20018 Donostia-San Sebastián, Spain.

[2] Carbon Bionanotechnology Group, Center for Cooperative Research in Biomaterials (CIC biomaGUNE), Basque Research and Technology Alliance (BRTA), 20014 San Sebastian, Spain.

[3] IIS Biodonostia, Neurosciences Area, Group of Neuromuscular Diseases. Paseo Dr. Begiristain s/n, 20014 San Sebastian, Spain.

[4] Panaxium SAS, Aix-en-Provence 13100, France.

[5] Ikerbasque, Basque Foundation for Science, 48013 Bilbao, Spain.

Corresponding author's emails: david.mecerreyes@ehu.es; miryam.criado@ehu.eus

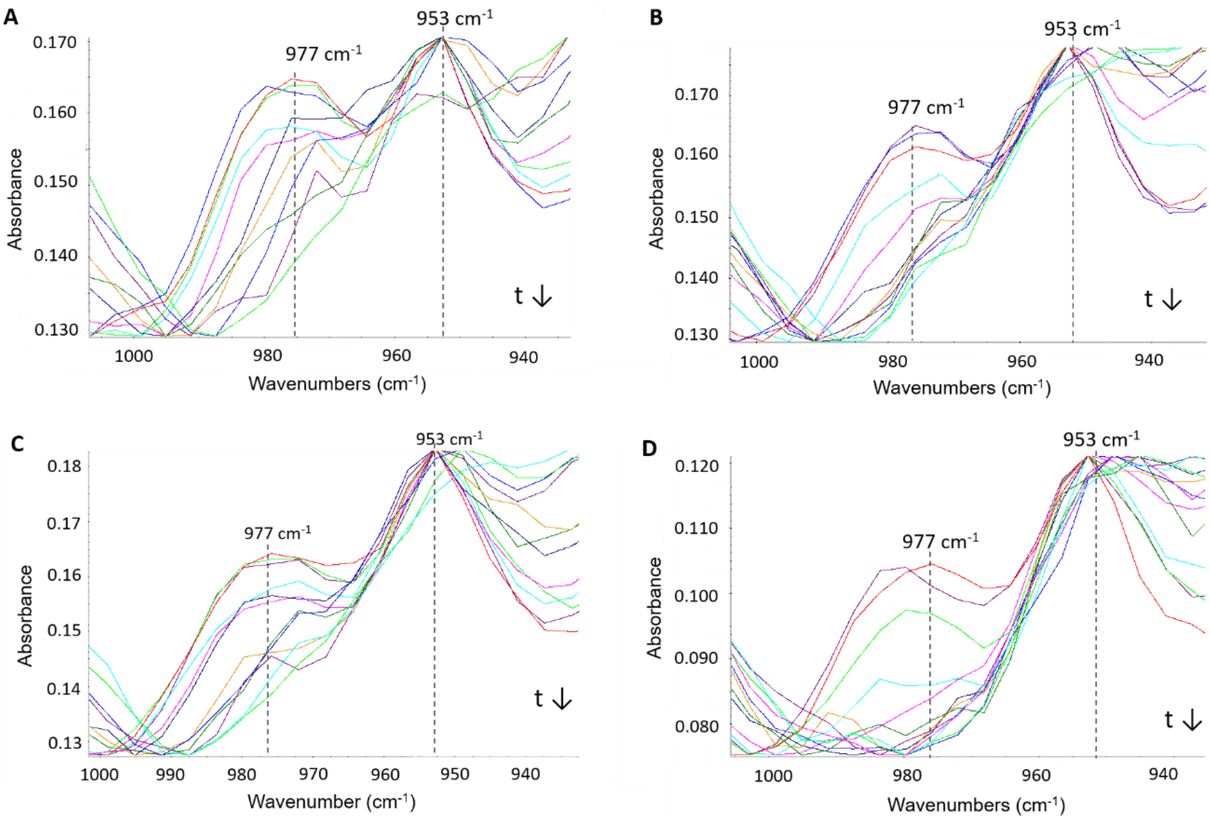

Figure S1. A) ATR-FTIR spectra of A) $PEDOT_{0.65}$_$PEGDA_{250}$, B) $PEDOT_{0.65}$_$PEGDA_{575}$, C) $PEDOT_{0.65}$_$PEGDA_{700}$, and D) $PEDOT_{0.00}$_$PEGDA_{700}$ inks in the C=C range at different times during the photo-polymerization process.

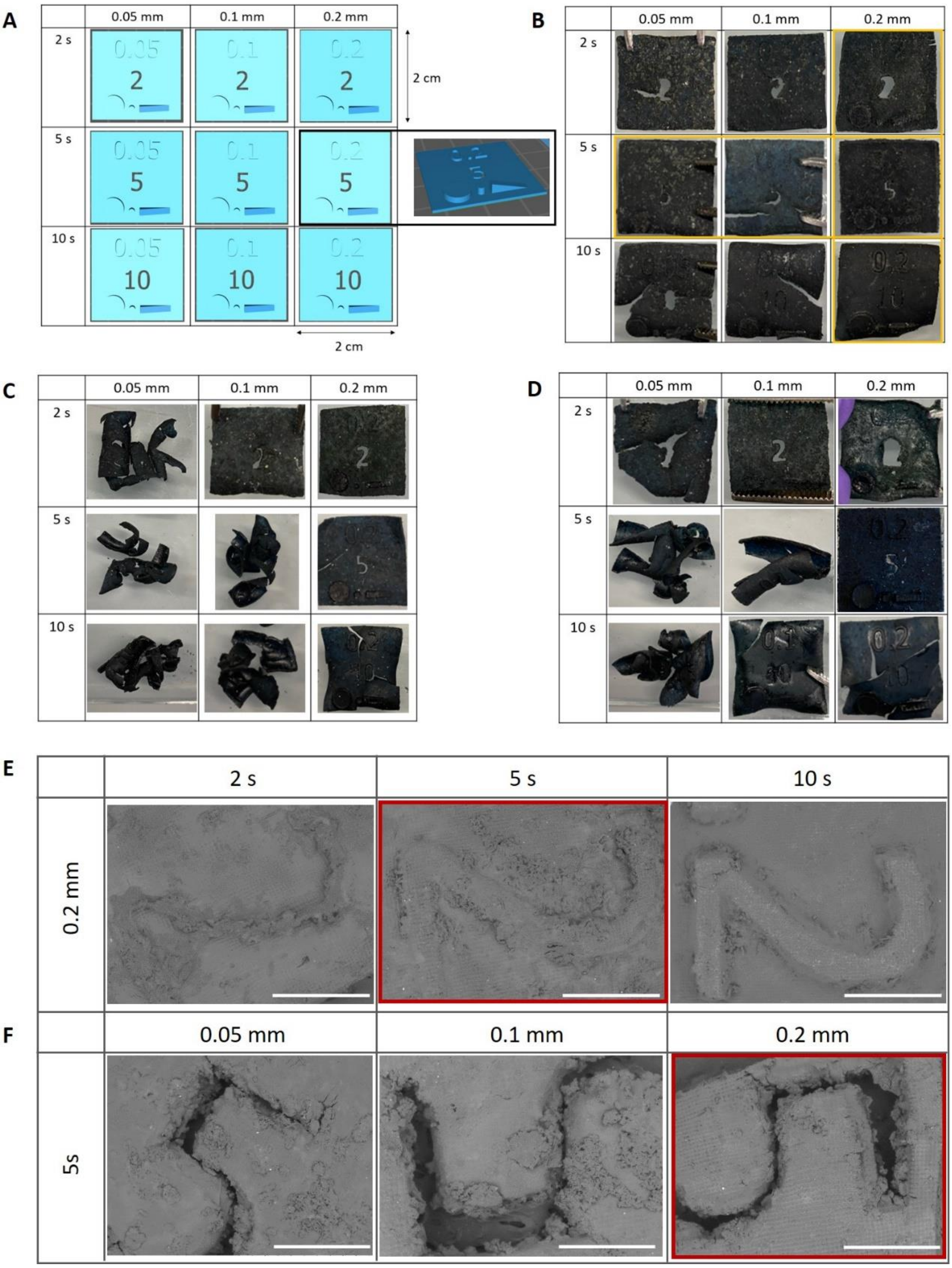

Figure S2. A) 3D patterns designed with Autodesk Inventor (Square Base = 2 mm side × 0.6 mm thickness). Photographs of B) $PEDOT_{0.65}$_$PEGDA_{250}$, C) $PEDOT_{0.65}$_$PEGDA_{575}$, and D) $PEDOT_{0.65}$_$PEGDA_{700}$ printed hydrogels varying the layer height and irradiation time. SEM images of $PEDOT_{0.65}$_$PEGDA_{250}$ printed hydrogels at: E) different irradiation times at a fixed layer height of 0.2 mm, and F) different layer heights and a fixed irradiation time of 5 seconds. Red rectangles show the obtained figures at optimum printing conditions (5 s, 0.2 mm). Scale bars = 2 mm.

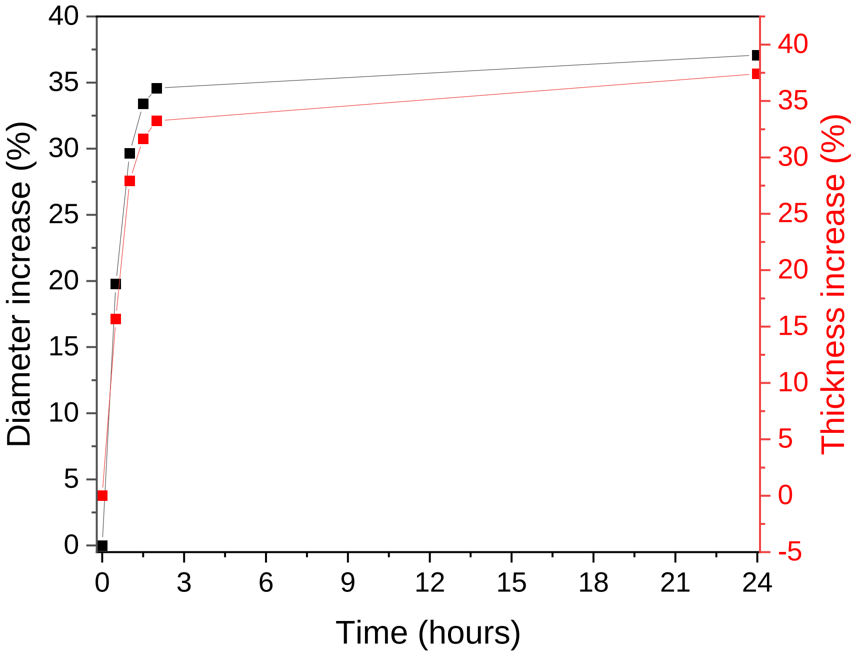

Figure S3. Evolution of the diameter and thickness during the swelling process.

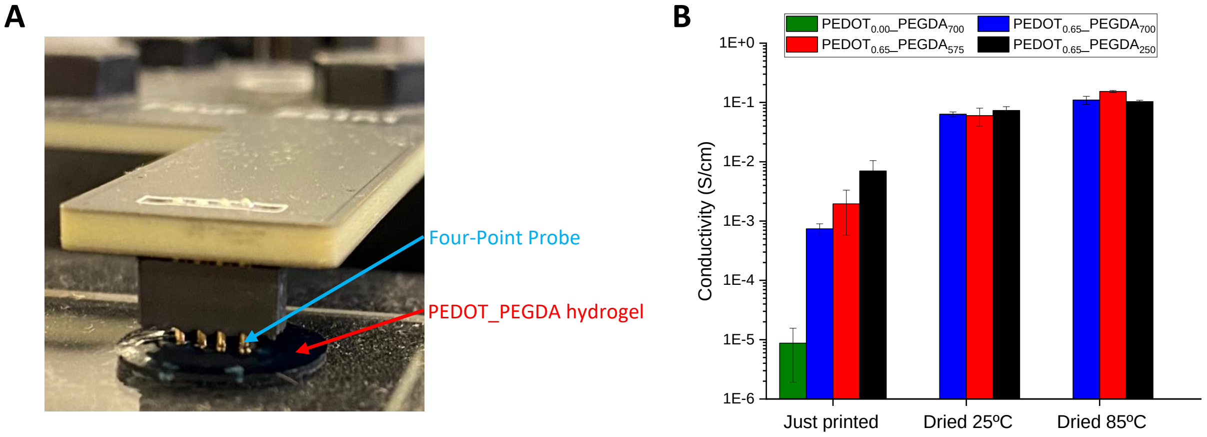

Figure S4. A) Picture of the electrical conductivity measurement of a PEDOT_PEGDA hydrogel by using the four-point probe equipment. B) Electrical conductivity of the hydrogels, PEDOT$_{0.00}$_PEGDA$_{700}$ (green bars), PEDOT$_{0.65}$_PEGDA$_{250}$ (black bars), PEDOT$_{0.65}$_PEGDA$_{575}$ (red bars), and PEDOT$_{0.65}$_PEGDA$_{700}$ (blue bars), dried at 25 and 85 °C.

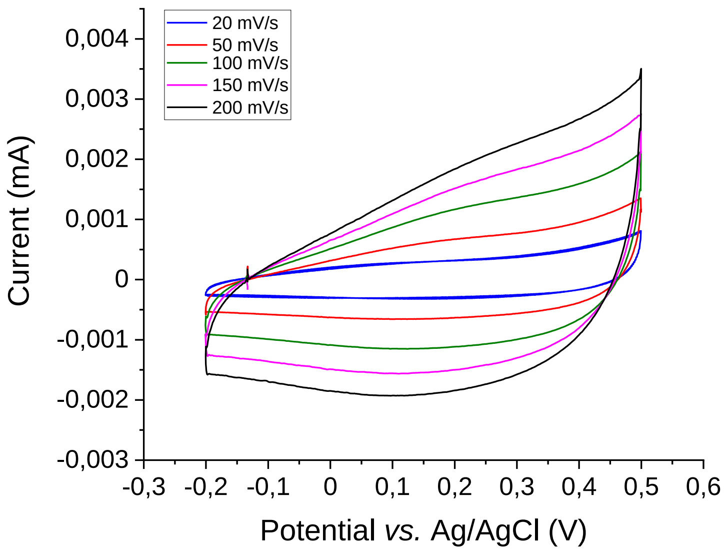

Figure S5. Cyclic voltammograms of the 3D printed hydrogel $PEDOT_{0.65}$_$PEGDA_{700}$ in 0.1M NaCl aqueous solution at different scan rates: 20 mV/s (blue curve), 50 mv/s (red curve), 100 mV/s (green curve), 150 mV/s (pink curve), and 200 mV/s (black curve).

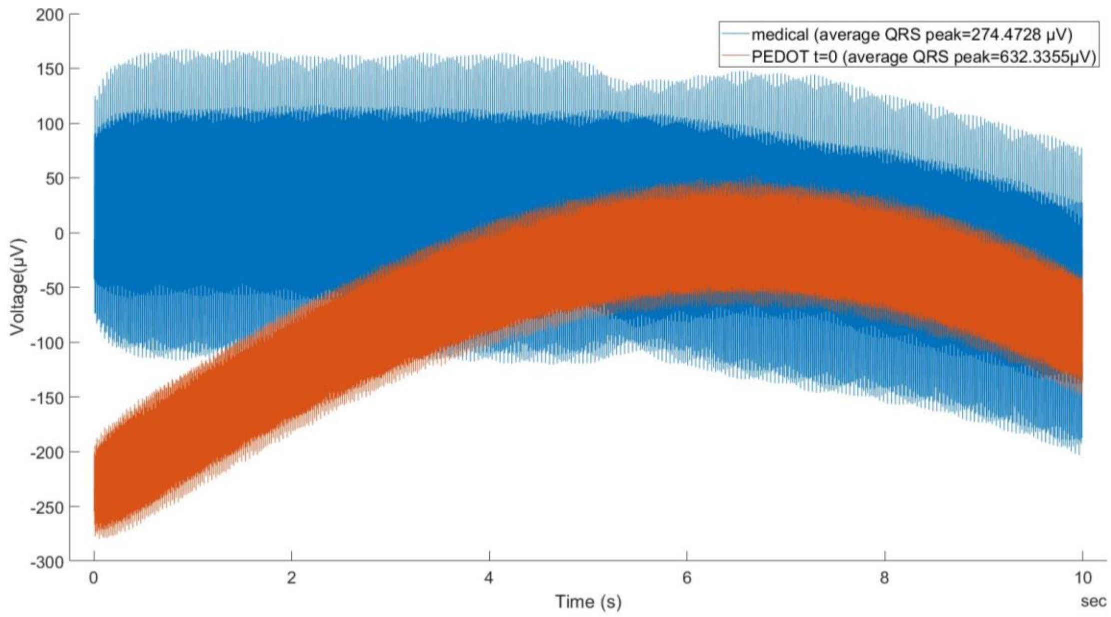

Figure S6. Signal to noise ratio of the electrodes built up with the $PEDOT_{0.65}$_$PEGDA_{700}$ electrodes (orange curve) and the standard medical electrode (blue curve). The average value of the peak of the QRS over 10 seconds is included in the legend.

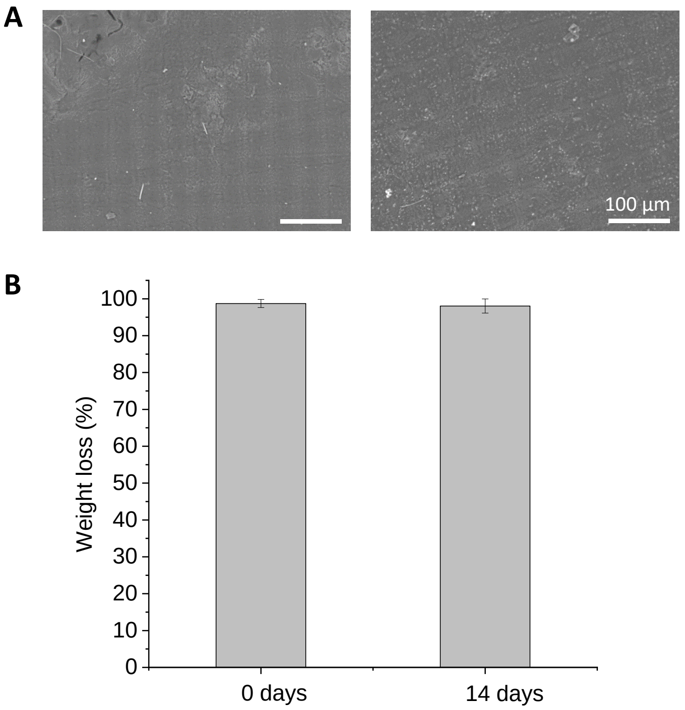

Figure S7. A) SEM images of the samples before and after 2 weeks in the wet state. B) Weight loss of samples after 2 weeks in swelling conditions.

Table S1. Nominal and real dimensions of the printed hydrogels.

Axis	Nominal size (mm)	Printing size (mm)		
		$PEDOT_{0.65}\_PEGDA_{700}$	$PEDOT_{0.65}\_PEGDA_{575}$	$PEDOT_{0.65}\_PEGDA_{250}$
x	20	20	20	20
y	20	20	20	20
z	0.53	0.49	0.47	0.67

Video S1:

https://drive.google.com/drive/folders/1LBQ0s8DfQ6L3gHJ1Sckaeyb7oB9XRutD
